# Size and Shape’s Effects on the High-Pressure Behavior of WS_2_ Nanomaterials

**DOI:** 10.3390/ma15082838

**Published:** 2022-04-12

**Authors:** Lei Yue, Dan Xu, Ziyu Wei, Tingting Zhao, Tao Lin, Reshef Tenne, Alla Zak, Quanjun Li, Bingbing Liu

**Affiliations:** 1 State Key Laboratory of Superhard Materials, Jilin University, Changchun 130012, China; yuelei19@mails.jlu.edu.cn (L.Y.); xudan@jlu.edu.cn (D.X.); weizy17@mails.jlu.edu.cn (Z.W.); ztt19@mails.jlu.edu.cn (T.Z.); lintao18@mails.jlu.edu.cn (T.L.); liubb@jlu.edu.cn (B.L.); 2 Department of Materials and Interfaces, Weizmann Institute, Rehovot 76100, Israel; 3 Faculty of Sciences, HIT—Holon Institute of Technology, 52 Golomb St., Holon 5810201, Israel; alzak@hit.ac.il

**Keywords:** high pressure, nanomaterials, WS_2_, crystal structure

## Abstract

Exploring the behavior of nanocrystals with varying shapes and sizes under high pressure is crucial to understanding the relationship between the morphology and properties of nanomaterials. In this study, we investigated the compression behaviors of WS_2_ nanotubes (NT-WS_2_) and fullerene-like nanoparticles (IF-WS_2_) by in situ high-pressure X-ray diffraction (XRD) and Raman spectroscopy. It was found that the bulk modulus of NT-WS_2_ is 81.7 GPa, which is approximately twice as large as that of IF-WS_2_ (46.3 GPa). This might be attributed to the fact that IF-WS_2_ with larger d-spacing along the c-axis and higher defect density are more compressible under isotropic pressure than NT-WS_2_. Thus, the slender NT-WS_2_ possess a more stable crystal structure than the IF-WS_2_. Our findings reveal that the effects of morphology and size play crucial roles in determining the high-pressure properties of WS_2_ nanoparticles, and provide significant insight into the relationship between structure and properties.

## 1. Introduction

Previous high-pressure investigations of nanomaterials have generated considerable interest owing to the emergence of the intriguing properties of these materials under extreme conditions [1,2,3,4]. Compared with conventional bulk materials, the effects of size and shape have been considered as two key factors determining the high-pressure behavior of nanoscale materials besides crystal structures [5]. Previous researches have revealed a variety of interesting phenomena. These include, among others, an increase in the phase transition pressure with decreasing particle size for CeSe nanoparticles [6], and shape-tuned, high-pressure phase transition sequences for TiO_2_ nanowires [7] and ZnS nanorods [8]. However, the effects of morphology and size on the high-pressure behavior of nanomaterials differ from one system to another. Thus, there is a further need to explore the effect of high pressure on different nanomaterials as a function of size and shape, which is of great significance in understanding the physical and chemical properties of nanomaterials.

Transition-metal dichalcogenides (TMDs) are layered materials with the chemical formula MX_2_, where M is a transition metal element and X is a chalcogen element (S, Se, Te) [9,10,11]. Layered TMDs share a common X-M-X sandwich structure in each layer, with hexagonally arranged chalcogen atoms in two planes separated by a sheet of metal atoms. The adjacent layers are weakly bonded together, through van der Waals forces, to form crystals in different polymorphs, with variations in stacking mode and metal-atom coordination [12,13,14]. WS_2_ is a member of the TMDs family and has attracted extensive attention owing to its structural [15], thermal [16], and optoelectronic [17] properties. In recent years, rapid progress has been made in precisely controlling the size and shape of synthesized WS_2_ nanocrystals with various methods, such as hydrothermal methods [18], liquid-phase exfoliation [19], pulsed lasers [20], and mechanical activation [21].

In particular, WS_2_ nanotubes (NT-WS_2_) and fullerene-like nanoparticles (IF-WS_2_) have been synthesized successfully. With their particular nested structure and tiny size, NT-WS_2_ exhibited intriguing photovoltaic and other optical properties [22,23]. IF-WS_2_ nanoparticles showed superior mechanical stability and solid-state lubrication qualities [24]. These structure-related properties have inspired significant interest in high-pressure investigations of these nanoparticles. IF-WS_2_ show very high compliance and shock-absorbing ability, which enables them to withstand shock pressure of up to 25.0 GPa with mild nanostructural degradation [25]. WS_2_ nanotubes exhibit structural breakdown perpendicular to the tube direction under high pressure, as well as macroscopic conductivity above the percolation threshold of 9.0 GPa [26]. However, high-temperature syntheses of nanomaterials cannot be easily controlled and, hence, structural and chemical defects are abundant. Therefore, crystal structure information is essential to a deeper comprehension of the pressure-property relationship. For this purpose, the structure and associated mechanical properties of nanomaterial systems under pressure can be investigated by high-pressure XRD using diamond anvil cell (DAC). Through the in situ structure and property characterization of nanomaterials, significant structure-property relationships can be depicted.

Here, we report a systematic study of WS_2_ nanoparticles with two contrasting particle shapes using in situ high-pressure XRD and Raman spectroscopy. We found that the compressibility of IF-WS_2_ and NT-WS_2_ varied significantly. The different high-pressure behavior of the IF-WS_2_ and NT-WS_2_ is discussed in terms of the effects of size and shape.

## 2. Experimental Section

The NT-WS_2_ and IF-WS_2_ used in this study were synthesized by a bottom-up solid–gas reaction [27,28], and the detailed growth mechanisms are presented in Appendix A.2. The samples were analyzed by transmission electron microscopy (TEM) and high-resolution transmission electron microscopy (HRTEM) using JEOL JEM-3010 (JEOL, Tokyo, Japan). High pressure was generated by utilizing a diamond anvil cell with a 500-micrometer culet size, and silicone oil was used as a pressure transfer medium. The gasket material used in the experiments was T301 steel, which was first pre-compressed to a thickness between 40 and 60 μm and then laser-drilled in the center to form a 180-micrometer diameter sample chamber. The pressure applied to the sample was determined by the ruby fluorescence method [29,30,31]. In situ high pressure XRD analyses were performed at room temperature using the RIGAKU Synergy Custom FR-X (RIGAKU, Tokyo, Japan), with an incident beam wavelength of 0.7107 Å. Rietveld refinement of the XRD data was accomplished utilizing the General Structure Analysis System (GSAS) [32]. Raman measurements were recorded using a LabRAM HR Evolution Instrument (HORIBA, Tokyo, Japan) with a 473-nanometer laser excitation source at room temperature.

## 3. Results and Discussion

**Figure 1:**
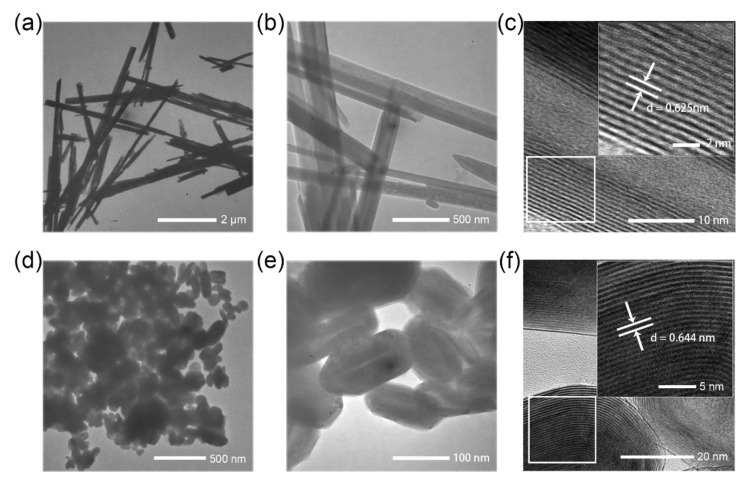
(**a**,**b**) TEM images of NT-WS_2_ at different magnifications. (**c**) HRTEM image of a typical NT-WS_2._ (**d**,**e**) TEM images of IF-WS_2_ at different magnifications. (**f**) HRTEM image of IF-WS_2_.

Figure 1 depicts the size and shape analyses of the two types of WS_2_ nanoparticle used in this work. Both kinds of nanoparticle have hollow cores a few nm in diameter. As seen in Figure 1a,b, the length of the NT-WS_2_ is about several micrometers and the diameter is in the range of 70–110 nm. The HRTEM image of a typical NT-WS_2_ in Figure 1c shows that it is multi-walled with an interlayer spacing of 6.25 Å between adjacent layers, corresponding to the (002) crystal plane. For comparison, Figure 1d,e shows the TEM images of the IF-WS_2_ at different magnifications. The dimensions of these hollow nanoparticles ranged from 60 to 130 nm in size. As shown in the HRTEM image of Figure 1f, the quasi-nanosphere IF nanoparticle consisted of approximately 30 closed WS_2_ layers in an onion-like nested arrangement, and the distance between the adjacent layers was 6.44 Å, corresponding to the (002) crystal plane. This expansion is attributable to the excessive strain in the vicinity of the bending point of the layers. In the less-curved and flatter areas, the interlayer spacing was shorter but was nevertheless larger than in the 2H-WS_2_ flakes (6.18 Å).

**Figure 2:**
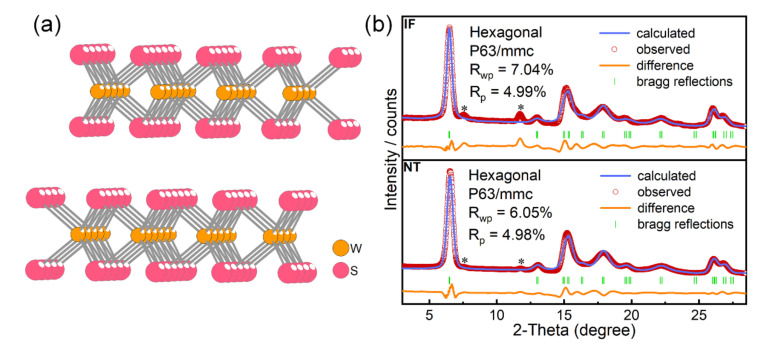
(**a**) Crystal-structure model of layered 2H-WS_2_. (**b**) Rietveld refinements plot of NT-WS_2_ and IF-WS_2_ at atmospheric pressure in space group P63/mmc. Peaks marked with an asterisk are the unconverted WO_X_ phases, possibly W_18_O_49_ or WO_2_.

The XRD analysis revealed that both kinds of nanoparticle adopted a hexagonal structure with the space group P63/mmc. As illustrated in Figure 2a, the WS_2_ featured a layered structure, with tungsten atoms sandwiched between two sheets of sulfur atoms (S−W−S) in each layer. Each tungsten atom is covalently bonded to three sulfur atoms in the lower plane and three sulfur atoms in the upper sulfur plane in a trigonal prismatic coordination. The adjacent layers were stacked together by weak van der Waals interactions. Figure 2b shows XRD patterns of the two types of WS_2_ nanoparticle at ambient pressure (for the original patterns, see Figure A2). The overall peak position of the NT-WS_2_ shifted to a higher angle compared with the IF-WS_2_. Furthermore, the lattice constants obtained from the Rietveld refinement of the XRD data were a = b = 3.1900 Å, c = 12.8614 Å, and V = 113.350 Å^3^ for the IF-WS_2_ and a = b = 3.1525 Å, c = 12.5576 Å, and V = 108.083 Å^3^ for the NT-WS_2_. The IF-WS_2_ had larger a and c lattice parameters than the NT-WS_2_ because they were more strained. Table 1 summarizes the results of the XRD analysis and compares them to those of the bulk 2H-WS_2_. It can be concluded from the table that the lattice parameters of the folded layers for both nanotubes and IF nanoparticles were larger than those of the bulk 2H-WS_2_. This expansion has been discussed in the past [27,33] and is attributable to the strain of the folded layers. The fact that the IF nanoparticles showed larger lattice expansion compared to the nanotubes is attributable to the fact that in the former, the layers were folded along two axes (a and b), which induced a large strain, while the nanotubes were folded along one axis (a or b, or a combination of the two axes for chiral nanotubes) only.

**Figure 3:**
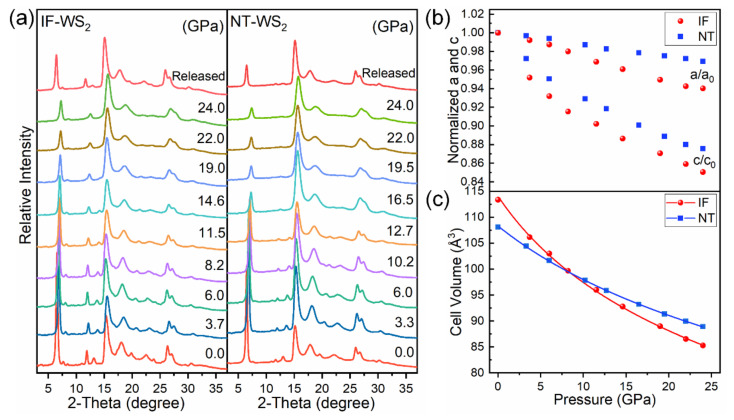
(**a**) Typical XRD patterns of IF-WS_2_ and NT-WS_2_ under different pressures. (**b**) Normalized cell parameters a/a_0_ and c/c_0_ versus pressure. (**c**) The volume of IF-WS_2_ and NT-WS_2_ versus pressure, solid lines represent the results of the fitting to the equations of state.

**Figure A1:**
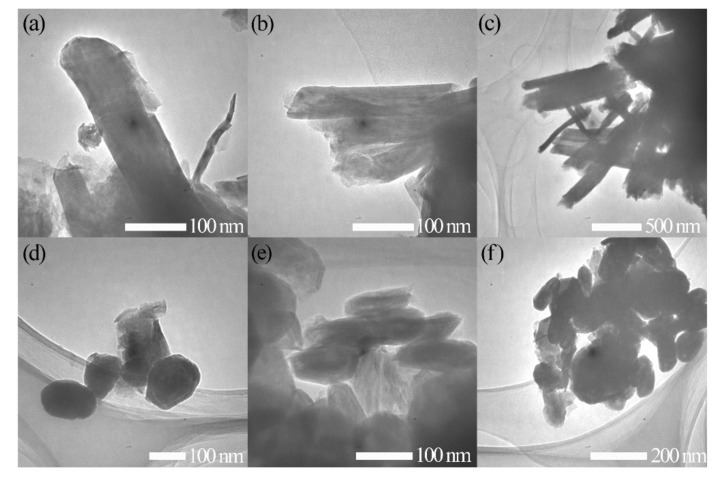
(**a**–**c**) TEM image of the released NT-WS_2_ after compression to 24.0 GPa. (**d**–**f**) TEM image of the released IF-WS_2_ after compression to 24.0 GPa.

**Figure A2:**
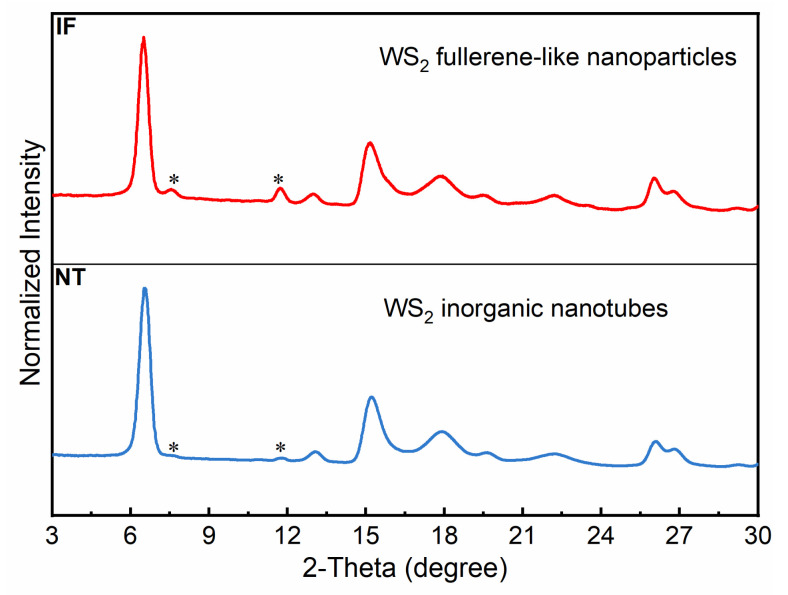
XRD pattern of NT-WS_2_ and IF-WS_2_ at ambient pressure. Peaks marked with an asterisk are W_18_O_49_ or WO_2_.

To detect the pressure-induced structural evolution, high-pressure XRD measurements of the NT-WS_2_ and IF-WS_2_ were carried out up to 24.0 GPa. Figure 3a presents the typical XRD patterns of the IF-WS_2_ and NT-WS_2_ under various pressure conditions. As the pressure increased, the peak positions moved toward higher angles, reflecting the pressure-driven contraction of the unit cell, accompanied by the merging, broadening, and weakening of some peaks. All the patterns matched well with the hexagonal structure (JCPDS no. 08-0237) and no phase transition occurred up to 24.0 GPa. Upon decompression, this trend was reversible for both kinds of sample. In addition, the WS_2_ nanotubes broke into several pieces when decompressed to ambient conditions, while the quasi-spherical IF-WS_2_ were somewhat squashed under the pressure and also partially exfoliated (see Figure A1). The morphology change in the NT-WS_2_ was consistent with previous work [26]. The refined lattice constants and the unit cell volume versus pressure are depicted in Figure 3b,c, respectively. Upon applying 24.0 GPa, the length of the lattice constants along the c- and a-axes decreased by 12% and 3% for the NT-WS_2_, compared with 15% and 6% for the IF-WS_2_. For all cases, the c-axis length was more compressible than the a-axis length owing to the weak van der Waals forces between the adjacent layers. Furthermore, the covalent bonds (in the a–b plane) were angle-sensitive, less tolerant to distortions and, hence, less compressible. Additionally, the relative length reduction of the a and the c lattice parameters was more pronounced in the IF-WS_2_, resulting in the overall volume compressibility being larger than that of NT-WS_2_. Furthermore, we employed a second-order Birch-Murnaghan equation of state to fit the volume-pressure relationship [34]. After fitting the data, the obtained bulk moduli of the IF-WS_2_ and NT-WS_2_ were 46.3 GPa and 81.7 GPa, respectively. Obviously, the latter showed a larger bulk modulus than the former, indicating that the NT-WS_2_ were less compressible than the IF-WS_2_. The bulk 2H-WS_2_ was reported to have a bulk modulus of 56.7 GPa [35] or 63.0 GPa [36]. Thus, the order of the bulk moduli for the WS_2_ was NT-WS_2_ > Bulk WS_2_ > IF-WS_2_. Similar trends were also observed in ZnO [7] and CdS nanomaterials [2]; these were mainly attributed to differences in the shape and size of the materials. The fact that the bulk modulus of the nanotubes was larger than that of the bulk material is intriguing; it was probably due to the closed-layer morphology of the nanotube, which provided extra elasticity.

**Figure 4:**
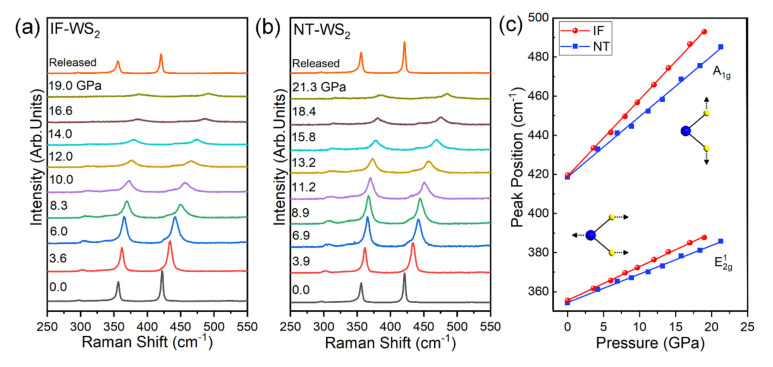
(**a**) Raman measurements of IF-WS_2_ at typical pressures up to 19.0 GPa. (**b**) Raman measurements of NT-WS_2_ at typical pressure up to 21.3 GPa. (**c**) Phonon frequencies of NT-WS_2_ and IF-WS_2_ as a function of pressure.

Raman spectra can be directly correlated with the compression rate through the Grüneisen parameter [37,38,39]. To further validate the different compression ratios between the IF-WS_2_ and the NT-WS_2_, we performed high-pressure Raman spectra measurements up to 19.0 GPa and 21.3 GPa, respectively. The results are shown in Figure 4a,b. At ambient conditions, two obvious Raman bands were observed at 355.5 cm^−1^ and 419.5 cm^−1^ for the IF-WS_2_ and, correspondingly, two bands at 354.2 cm^−1^ and 418.5 cm^−1^ for the NT-WS_2_. These two Raman active modes were assigned as $E_{2g}^{1}$ and A_1g_ symmetry modes, respectively [26,40].

**Table 1:** Lattice parameters a and c and cell volume V of three types of WS_2_ at ambient pressure.

Samples	a = b [Å]	c [Å]	V [Å^3^]	K_0_ (GPa)	Reference
Bulk WS_2_	3.138	12.416	105.870	56.7	Theoretical [33]
Bulk WS_2_	3.158	12.375	107.450	63.0	Experimental [34]
NT-WS_2_	3.153	12.558	108.083	81.7	This work
IF-WS_2_	3.190	12.861	113.350	46.3	This work

**Figure A3:**
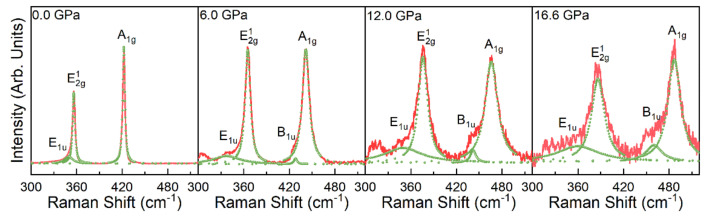
The Lorentzian fit curves of IF-WS_2_ under various pressures.

**Figure A4:**
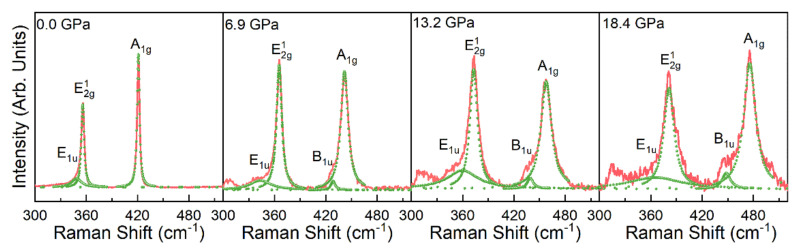
The Lorentzian fit curves of NT-WS_2_ under various pressures.

**Figure A5:**
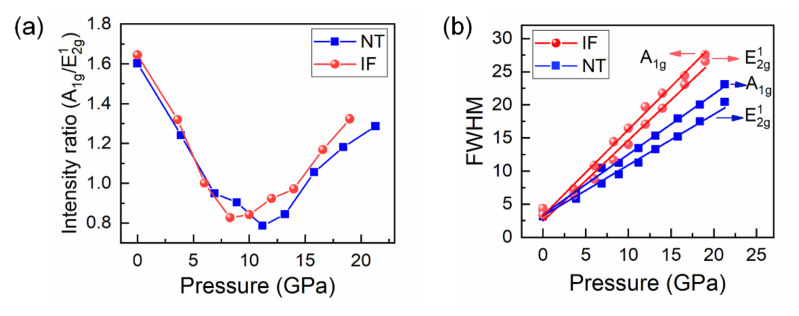
(**a**) Pressure-dependent intensity ratio of A_1g_ and $E_{2g}^{1}$ modes. (**b**) Pressure-dependent FWHM of A_1g_ and $E_{2g}^{1}$ modes for NT-WS_2_ and IF-WS_2_.

Notably, the two modes of the IF-WS_2_ were somewhat higher in frequency than the NT-WS_2_. This result is compatible with the larger interlayer spacing in the IF-WS_2_ compared to the NT-WS_2_. It is attributable to the larger built-in strain in the IF-WS_2_, and the folding of the layers along two axes rather than the single folding axis in the NT-WS_2_. Upon compression, all the Raman modes shifted monotonically and continuously to higher frequencies; no apparent phase transition was observed during the studied pressure regions, which agrees with the high-pressure XRD results. Combining the peak position and pressure data of the two samples in Figure 4c highlights a rather interesting trend. The solid line represents the least-squares fit to the experimental data generated by a pressure-dependent linear-fit function:

E(P) = E_0_ + αP (1)

where E_0_ is the Raman peak position at ambient pressure and α is the pressure coefficient. The best fit to the data yields phonon frequency pressure coefficients α. In the case of the $E_{2g}^{1}$ symmetry mode, the pressure coefficients α are 1.72 and 1.46 meV·GPa^−1^ for the IF-WS_2_ and NT-WS_2_, respectively. For the A_1g_ symmetry mode, the α are 3.91 and 3.10 meV·GPa^−1^ for the IF-WS_2_ and NT-WS_2_, respectively. In comparison, the IF-WS_2_ phase has greater pressure coefficients in both vibration modes, indicating a higher sensitivity to pressure. The varied compressibility between the two samples was probably responsible for the different pressure coefficients in the Raman modes. These results further confirm that IF-WS_2_ is more compressible than NT-WS_2_. To conduct a further analysis of the Raman spectra, we fitted the spectra of the NT-WS_2_ and IF-WS_2_ with Lorentzian profiles, as seen in Figure A3 and Figure A4. By fitting the data, we obtained information on the position, intensity, and FWHM (full width at half-maximum) of each Raman vibration mode. In the low-pressure region, the intensity ratio of the A_1g_ and $E_{2g}^{1}$ modes (I (A_1g_)/I ($E_{2g}^{1}$)) decreased but increased again after going through a minimum of around 8.3 GPa and 11.2 GPa for the IF-WS_2_ and NT-WS_2_, respectively. A similar trend was also observed in the W/WS_2_ fullerene-like nanospheres [41]. The effect of the pressure on the sample was anisotropic, inducing the shape transformation and the associated changes in the curvature of the sample, even under quasi-hydrostatic pressure, which explains this change in the intensity ratio. Furthermore, the FWHM broadened almost linearly as the pressure increased, which may have been due to the reduced crystallinity caused by the pressure. However, there was no distinct mutation phenomenon in the FWHM, demonstrating that no structural phase transition occurred in the studied pressure range (see Figure A5).

Here, we discuss the possible reasons for the difference in compressibility between the NT-WS_2_ and the IF-WS_2_. Our results indicate that the order of their compressibility is NT-WS_2_ < Bulk WS_2_ < IF-WS_2_. Numerous studies suggested that nanomaterials undergo phase transitions at higher pressures as the average particle size decreases and exhibit higher bulk moduli than those of their corresponding bulk materials. This trend can be explained by the increased surface energy with the increasing surface-to-volume ratio of nanoparticles [42,43]. The slender shape of the NT-WS_2_ entails a higher surface-to-volume ratio compared to bulk materials, which provides increased hardness. However, the bulk modulus of the IF-WS_2_ displayed the opposite result, i.e., its modulus was smaller than that of the bulk 2H-WS_2_ flakes. We suggest that this discrepancy may have arisen from the particle morphology and the relatively large defect density in the IF-WS_2_. More specifically, IF-WS_2_ are quasi-nanosphere nanoparticles consisting of multiple closed WS_2_ layers in an onion-like nested arrangement. It is well known that owing to the weak van der Waals force between the adjacent layers, WS_2_ is more compressible along the c-axis than along the a-axis. Therefore, IF-WS_2_ displays higher c-axis compressibility. Moreover, perfect seaming of the folded layers along two directions (a- and b-axes) is not possible, leaving many defects in the IF’s structure. The fact that the nanotubes are folded along one axis only implies that they can more easily fold and seam and, hence, that their defect density is appreciably smaller than that of IF nanoparticles. This high defect density makes IF nanoparticles more prone to structural distortion under pressure. Another important factor is the interlayer shear. The uniaxial compression of individual WS_2_ nanotubes, which also involves interlayer shearing, was studied previously [44]. The interlayer shear modulus was found to be about 2.0 GPa. Under hydrostatic pressure, the layers also shear with respect to each other, which is an important strain relaxation mechanism for the nanotubes. In the case of the IF-WS_2_, the layers are interlocked and their ability to shear with respect to each other is more limited; hence, their ability to absorb strain is lower compared to the NT-WS_2_. Through the above discussion, we believe that our study provides a reference to better understand the effects of size and morphology on the high-pressure behavior of WS_2_ nanoparticles.

## 4. Conclusions

In summary, we performed in situ high-pressure XRD and Raman spectroscopy to investigate the high-pressure behavior of WS_2_ nanotubes (NT-WS_2_) and fullerene-like nanoparticles (IF-WS_2_). The results show that the structures of NT-WS_2_ and IF-WS_2_ remain stable up to 24.0 GPa, showing excellent compression-resisting behavior, which demonstrates their great potential to withstand very high applied loads when used as lubricants. The bulk moduli of the IF-WS_2_ and NT-WS_2_ were 46.3 GPa and 81.7 GPa, respectively, compared to ≈60 GPa for the bulk material, suggesting a remarkable difference in the compressibility of the samples. It is proposed that the crystal growth orientation, surface energy, defect density, interlayer shearing, and nanosize effects play a significant role in the high-pressure properties of these nanoparticles. Our work provides critical insights for the comprehension of structure-property relationship, which will be valuable in the design and development of novel functional nanomaterials.

## Data Availability

The data that support the findings of this study are available from the corresponding author upon reasonable request.

## Appendix A

### Appendix A.2. Synthesis of NT-WS_2_ and IF-WS_2_

The WS_2_ nanotubes (NT-WS_2_) were synthesized from the combined sulfurization-reduction process through the tungsten oxide precursor via vapor-gas-solid reaction (VGSR). Briefly, the nanosized spherical precursors of non-volatile tungsten oxide were reduced by hydrogen (H_2_) in various steps to produce a volatile tungsten suboxide phase, which subsequently became the building material for the growth of the oxide nano whiskers. In turn, the reaction of these whiskers with hydrogen sulfide (H_2_S) led to their gradual sulfurization, starting at the surface and in the middle of the oxide nano whiskers, to form hollow multi-walled WS_2_ nanotubes. This multi-step reaction took place in a one-pot process (the same reaction parameters for each step) at elevated temperatures of 840 °C in the reducing atmosphere of H_2_/H_2_S gases using N_2_ as a carrier gas as well. A horizontal reactor with a specially designed porous quartz reaction cell created the proper quasi-static conditions for the reaction, which involved sublimation.

The synthesis of the WS_2_ fullerene-like nanoparticles (IF-WS_2_ NPs) was similar to the nanotubes. In the case of the IF-WS_2_, spherical nanoparticles were prepared separately and under different reaction conditions. During the sulphidation process, H_2_S and H_2_ gases reacted rapidly with the surface of the hemispherical oxide NPs, resulting in the rapid formation of a number of closed WS_2_ layers that encased the oxide core. The oxide core was then partially reduced to WO_3−x_. Subsequently, a slow diffusion-controlled reaction allowed the oxide nanoparticles to be converted to hollow sulphide nanoparticles by a reaction that consumed the tungsten oxide core from the surface inwards.

## References

[1] Bai F., Bian K., Huang X., Wang Z., Fan H. (2019). Pressure induced nanoparticle phase behavior, property, and applications. Chem. Rev..

[2] Meng L., Lane J.M.D., Baca L., Tafoya J., Ao T., Stoltzfus B., Knudson M., Morgan D., Austin K., Park C. (2020). Shape dependence of pressure-induced phase transition in CdS semiconductor nanocrystals. J. Am. Chem. Soc..

[3] Li J., Liu B., Dong J., Li C., Dong Q., Lin T., Liu R., Wang P., Shen P., Li Q. (2020). Size and morphology effects on the high pressure behaviors of Mn_3_O_4_ nanorods. Nanoscale Adv..

[4] San-Miguel A. (2006). Nanomaterials under high-pressure. Chem. Soc. Rev..

[5] Kumar S.K., Krishnamoorti R. (2010). Nanocomposites: Structure, phase behavior, and properties. Annu. Rev. Chem. Biomol. Eng..

[6] Chen C.-C., Herhold A.B., Johnson C.S., Alivisatos A.P. (1997). Size dependence of structural metastability in semiconductor nanocrystals. Science.

[7] Li Q., Cheng B., Yang X., Liu R., Liu B., Liu J., Chen Z., Zou B., Cui T., Liu B. (2013). Morphology-tuned phase transitions of anatase TiO_2_ nanowires under high pressure. J. Phys. Chem. C.

[8] Li Z., Liu B., Yu S., Wang J., Li Q., Zou B., Cui T., Liu Z., Chen Z., Liu J. (2011). The study of structural transition of ZnS nanorods under high pressure. J. Phys. Chem. C.

[9] Dong Q., Li Q., Li S., Shi X., Niu S., Liu S., Liu R., Liu B., Luo X., Si J. (2021). Structural phase transition and superconductivity hierarchy in 1T-TaS2 under pressure up to 100 GPa. npj Quantum Mater..

[10] Mak K.F., Shan J. (2016). Photonics and optoelectronics of 2D semiconductor transition metal dichalcogenides. Nat. Photonics.

[11] Singh E., Singh P., Kim K.S., Yeom G.Y., Nalwa H.S. (2019). Flexible molybdenum disulfide (MoS_2_) atomic layers for wearable electronics and optoelectronics. ACS Appl. Mater. Interfaces.

[12] Hu Z., Wu Z., Han C., He J., Ni Z., Chen W. (2018). Two-dimensional transition metal dichalcogenides: Interface and defect engineering. Chem. Soc. Rev..

[13] Mueller T., Malic E. (2018). Exciton physics and device application of two-dimensional transition metal dichalcogenide semiconductors. npj 2D Mater. Appl..

[14] Wen X., Gong Z., Li D. (2019). Nonlinear optics of two-dimensional transition metal dichalcogenides. InfoMat.

[15] Duerloo K.-A.N., Li Y., Reed E.J. (2014). Structural phase transitions in two-dimensional Mo-and W-dichalcogenide monolayers. Nat. Commun..

[16] Gandi A.N., Schwingenschlögl U. (2014). WS_2_ as an excellent high-temperature thermoelectric material. Chem. Mater..

[17] Ovchinnikov D., Allain A., Huang Y.-S., Dumcenco D., Kis A. (2014). Electrical transport properties of single-layer WS_2_. ACS Nano.

[18] Hu J. (2019). Preparation and Optical Absorption Properties of Tungsten Disulfide Nanomaterials. Acta Microsc..

[19] Wu F., Xia Y., Sun M., Xie A. (2018). Two-dimensional (2D) few-layers WS_2_ nanosheets: An ideal nanomaterials with tunable electromagnetic absorption performance. Appl. Phys. Lett..

[20] Hu J.J., Zabinski J.S., Sanders J.H., Bultman J.E., Voevodin A.A. (2006). Pulsed Laser Syntheses of Layer-Structured WS_2_ Nanomaterials in Water. J. Phys. Chem. B.

[21] Wu Z., Wang D., Zan X., Sun A. (2010). Synthesis of WS_2_ nanosheets by a novel mechanical activation method. Mater. Lett..

[22] Levi R., Bitton O., Leitus G., Tenne R., Joselevich E. (2013). Field-effect transistors based on WS_2_ nanotubes with high current-carrying capacity. Nano Lett..

[23] Zhang C., Wang S., Yang L., Liu Y., Xu T., Ning Z., Zak A., Zhang Z., Tenne R., Chen Q. (2012). High-performance photodetectors for visible and near-infrared lights based on individual WS_2_ nanotubes. Appl. Phys. Lett..

[24] Brown S., Musfeldt J., Mihut I., Betts J., Migliori A., Zak A., Tenne R. (2007). Bulk vs nanoscale WS_2_: Finite size effects and solid-state lubrication. Nano Lett..

[25] Zhu Y.Q., Sekine T., Li Y.H., Fay M.W., Zhao Y.M., Patrick Poa C., Wang W.X., Roe M.J., Brown P.D., Fleischer N. (2005). Shock-absorbing and failure mechanisms of WS_2_ and MoS_2_ nanoparticles with fullerene-like structures under shock wave pressure. J. Am. Chem. Soc..

[26] O’Neal K.R., Cherian J., Zak A., Tenne R., Liu Z., Musfeldt J. (2016). High pressure vibrational properties of WS_2_ nanotubes. Nano Lett..

[27] Zak A., Sallacan-Ecker L., Margolin A., Feldman Y., Popovitz-Biro R., Albu-Yaron A., Genut M., Tenne R. (2010). Scaling up of the WS_2_ nanotubes synthesis. Fuller. Nanotub. Carbon Nanostruct..

[28] Feldman Y., Frey G., Homyonfer M., Lyakhovitskaya V., Margulis L., Cohen H., Hodes G., Hutchison J., Tenne R. (1996). Bulk synthesis of inorganic fullerene-like MS_2_ (M= Mo, W) from the respective trioxides and the reaction mechanism. J. Am. Chem. Soc..

[29] Mao H., Bell P., Shaner J.T., Steinberg D. (1978). Specific volume measurements of Cu, Mo, Pd, and Ag and calibration of the ruby R 1 fluorescence pressure gauge from 0.06 to 1 Mbar. J. Appl. Phys..

[30] Ross N.L. (1997). The equation of state and high-pressure behavior of magnesite. Am. Mineral..

[31] Piermarini G.J., Block S., Barnett J., Forman R. (1975). Calibration of the pressure dependence of the R1 ruby fluorescence line to 195 kbar. J. Appl. Phys..

[32] Toby B.H. (2001). EXPGUI, a graphical user interface for GSAS. J. Appl. Crystallogr..

[33] Tenne R. (2006). Inorganic nanotubes and fullerene-like nanoparticles. J. Mater. Res..

[34] Katsura T., Tange Y. (2019). A simple derivation of the Birch-Murnaghan equations of state (EOSs) and comparison with EOSs derived from other definitions of finite strain. Minerals.

[35] Li L., Zeng Z.-Y., Liang T., Tang M., Cheng Y. (2017). Elastic Properties and Electronic Structure of WS_2_ under Pressure from First-principles Calculations. Z. Nat. A.

[36] Bandaru N., Kumar R.S., Baker J., Tschauner O., Hartmann T., Zhao Y., Venkat R. (2014). Structural stability of WS_2_ under high pressure. .Int. J. Mod. Phys..

[37] Del Corro E., de la Roza A.O., Taravillo M., Baonza V.G. (2012). Raman modes and Grüneisen parameters of graphite under compressive biaxial stress. Carbon.

[38] Proctor J.E., Gregoryanz E., Novoselov K.S., Lotya M., Coleman J.N., Halsall M.P. (2009). High-pressure Raman spectroscopy of graphene. Phys. Rev. B.

[39] Livneh T., Sterer E. (2010). Resonant Raman scattering at exciton states tuned by pressure and temperature in 2H-MoS_2_. Phys. Rev. B.

[40] Joly-Pottuz L., Martin J.-M., Dassenoy F., Belin M., Montagnac G., Reynard B., Fleischer N. (2006). Pressure-induced exfoliation of inorganic fullerene-like WS_2_ particles in a Hertzian contact. J. Appl. Phys..

[41] Yu S.D., Chang L.X., Yang H.B., Liu B.B., Hou Y.Y., Wang L., Yao M.G., Cui T., Zou G.T. (2007). Study of the hydrostatic pressure dependence of the Raman spectrum of W/WS_2_ fullerene-like nanosphere with core-shell structure. J. Phys. Condens. Matter.

[42] Qadri S.B., Yang J., Ratna B., Skelton E.F., Hu J. (1996). Pressure induced structural transitions in nanometer size particles of PbS. Appl. Phys. Lett..

[43] Tolbert S., Alivisatos A. (1994). Size dependence of a first order solid-solid phase transition: The wurtzite to rock salt transformation in CdSe nanocrystals. Science.

[44] Chen Y., Lai Z., Zhang X., Fan Z., He Q., Tan C., Zhang H. (2020). Phase engineering of nanomaterials. Nat. Rev. Chem..

